# Identification of Antimony- and Arsenic-Oxidizing Bacteria Associated with Antimony Mine Tailing

**DOI:** 10.1264/jsme2.ME12217

**Published:** 2013-05-11

**Authors:** Natsuko Hamamura, Koh Fukushima, Takaaki Itai

**Affiliations:** 1 Center for Marine Environmental Studies, Ehime University, Matsuyama 790–8577 Japan

**Keywords:** antinomy, arsenic, *aio*, arsenite oxidase, *Stenotrophomonas*

## Abstract

Antimony (Sb) is a naturally occurring toxic element commonly associated with arsenic (As) in the environment and both elements have similar chemistry and toxicity. Increasing numbers of studies have focused on microbial As transformations, while microbial Sb interactions are still not well understood. To gain insight into microbial roles in the geochemical cycling of Sb and As, soils from Sb mine tailing were examined for the presence of Sb- and As-oxidizing bacteria. After aerobic enrichment culturing with As^III^ (10 mM) or Sb^III^ (100 μM), pure cultures of *Pseudomonas-* and *Stenotrophomonas-*related isolates with Sb^III^ oxidation activities and a *Sinorhizobium-*related isolate capable of As^III^ oxidation were obtained. The As^III^-oxidizing *Sinorhizobium* isolate possessed the aerobic arsenite oxidase gene (*aioA*), the expression of which was induced in the presence of As^III^ or Sb^III^. However, no Sb^III^ oxidation activity was detected from the *Sinorhizobium-*related isolate, suggesting the involvement of different mechanisms for Sb and As oxidation. These results demonstrate that indigenous microorganisms associated with Sb mine soils are capable of Sb and As oxidation, and potentially contribute to the speciation and mobility of Sb and As *in situ*.

Antimony (Sb) is a naturally occurring toxic element and is considered to be a priority pollutant of interest by the USEPA (the maximum contaminant level in drinking water is 6 μg L^−1^). Although the concentrations of Sb in natural systems are generally low (less than 1 mg kg^−1^ in soil and 1 μg L^−1^ in surface waters [13]), elevated levels of Sb have been released via mining activities and other anthropogenic activities due to its increasing industrial use. In the environment, antimony is commonly associated with arsenic (As) and both elements exhibit similar geochemical properties and toxicological effects that depend on their chemical form and oxidation state. Antimony and arsenic can exist in four oxidation states (−III, 0, III and V), while they are mainly found in two oxidation states, trivalent (III) and pentavalent (V), in natural systems. Antimonate [Sb(V)] and arsenate [As(V)] are thermodynamically stable species in aerobic environments and occur primarily as H_2_AsO_4_^−^ and HAsO_4_^2−^, or Sb(OH)_6_^−^. In anaerobic environments, the dominant solution species of antimonite [Sb(III)] and arsenite [As(III)] occur as neutral Sb(OH)_3_^0^ and As(OH)_3_^0^ in the environmentally relevant pH range (14). As(III) and Sb(III) are highly reactive with thiol-containing proteins and are considered more toxic than As(V) and Sb(V) (13, 38).

Despite its toxicity, microorganisms have developed resistance mechanisms to tolerate As and some can utilize As for respiratory metabolism to gain energy for growth. As resistance mechanisms in bacteria typically involve As(V) reduction to As(III) by an arsenate reductase (ArsC) and As(III) is extruded by membrane-located ArsB efflux pump (15). In addition, dissimilatory arsenate reductase (Arr) has been identified in phylogenetially diverse groups of dissimilatory As(V)-respiring bacteria (34). Oxidation of As(III) coupled to O_2_ reduction is catalyzed by arsenite oxidase (Aio) and has been described in numerous heterotrophic bacteria (34) as well as in some chemoautotrophs, which can gain energy from As(III) oxidation for CO_2_ fixation (3, 9, 10, 31, 33). Anaerobic As(III) oxidation coupled to nitrate reduction or photosynthesis is carried out by another group of arsenite oxidases named ArxA, which appears to be evolutionally more closely related to Arr than to Aio (39, 40).

Although increasing numbers of studies have focused on microbial roles in As transformations, microbially-mediated Sb transformations are still not well understood. Possibly due to the structural similarities between As and Sb, some of the As-metabolizing mechanisms were also considered to process Sb. For instance, both As(III) and Sb(III) enter the cells via a glycerol facilitator, the GlpF uptake system, and are exported by the same ArsB system in bacteria (15, 28, 32). It was also shown that both As(III) and Sb(III) induced the expression of arsenic resistance *ars* operon (21) and the aerobic As(III) oxidase gene (*aio*) operon (8, 25). There have been only a few reports regarding microbial redox transformations of antimony. In the 70s, a couple of studies described the oxidation of senarmonite (Sb_2_O_3_) to Sb_2_O_5_ by a chemolithotrophic organism referred to as *Stibiobacter senarmontii* (reviewed in reference [11]). Recently, a study by Lehr *et al.* showed Sb(III) oxidation by As(III)-oxidizing *Agrobacterium tumefacience* and eukaryotic acido-thermophilic *Cyanidiales* alga isolate (25). Since Sb(III) oxidation was observed with two mutant strains of *A. tumefacience* incapable of As(III) oxidation, it was suggested that Sb(III) oxidation is catalyzed by a pathway different from the As(III) oxidation pathway catalyzed by Aio in this organism (25). To our knowledge, there have been no reports of other microorganisms capable of Sb oxidation, or further characterization of microbial Sb oxidation mechanisms.

To gain insight into microbial roles in the dynamics of Sb and As in soil water environment, we examined soils from antimony (stibnite: Sb_2_S_3_) mine tailing (Ichinokawa, Ehime, Japan) for the presence of Sb- and As-transforming bacterial populations. This report describes the isolation and characterization of novel Sb(III)-oxidizing bacteria along with an As(III)-oxidizing bacterium obtained from the mine soils.

## Materials and Methods

### Sample collection and chemical analysis

Soil samples were collected in October 2009 from Ichinokawa mine (Ehime, Japan) tailing areas (33°53′20.4″N, 133°12′51.6″E), which was formerly one of the largest Sb (stibnite: Sb_2_S_3_) mines in the world (6, 29). The sampling sites were located approximately 5 m downward from the mine pit (site 1) and 30 m to the side (site 2) of the mine pit. The soils were collected from four depth ranges (0–3, 3–6, 6–9, and 9–12 cm) successively from the surface layer using sterile techniques, transported on ice to the laboratory and stored at 4°C for cultivation and −20°C for geochemical analyses. The pH of the surface layer soils (0–3 cm) was determined in soil-water extracts (1:1) on site to be 7.2 and 5.9 for site 1 and 2, respectively. For geochemical characterization, soils were freeze-dried, passed through a 500-μm stainless steel sieve and homogenized. The soil samples were then digested in a mixture of HNO_3_ and HF using a microwave system and inorganic constituents were determined using an inductively coupled plasma mass spectrometer (ICP-MS; Agilent 7500cs, Agilent) as described previously (16). Analytical accuracy was confirmed by analyzing standard reference material NIST-2710 (NIST) and resulted in the recovery of the nine elements being 99.2±7.1% of certified values.

### Molecular analysis

For molecular analysis, soil samples were collected using sterile techniques, stored in RNAlater (Ambion, Austin, TX) immediately upon sampling, transported on ice and stored at −20°C after returning to the laboratory. Total DNA was extracted from soil samples using the PowerSoil DNA Isolation Kit (MoBio, Carlsbad, CA). For denaturing gradient gel electrophoresis (DGGE) analysis, 16S rRNA gene fragments were PCR-amplified using *Bacteria-*specific primer 1070F and the universal primer 1392R containing a GC-clamp, followed by separation of PCR products using DGGE as described previously (18). The reproducibility of the techniques was confirmed using DNA extracts prepared in duplicate from soil samples (Fig. S1, site 1). Soil DNA extracts were also screened for arsenite oxidase genes (*aioA*, *arxA*) and dissimilatory arsenate reductase genes (*arrA*) by PCR amplification using the primers and conditions described previously: for *aioA*; aroA95f and aroA599r (19), for *arxA*; arxA_Deg_F_B and arxA_Deg_R_B (40), for *arrA*; HAArrA-D1F and HAArrA-G2R (23) and ArrAfwd and ArrArev (27). Amplified products were analyzed by electrophoresis in 1.5% agarose gels. Cells from enrichment cultures and isolates were grown in liquid media as described below, collected by centrifugation, and subjected to DNA extraction followed by 16S rRNA gene amplification for DGGE and PCR screening for the arsenic transformation genes as described above. Dominant bands in the DGGE gels were purified and sequenced as described previously (12). Near full-length 16S rRNA genes were also determined for the obtained isolates as described previously (18). Sequences were assembled using Sequencher 4.1 (Gene Codes Corporation, Ann Arbor, MI) and compared to the GenBank database using BLAST (1). Phylogenetic analysis was conducted using MEGA version 5 software (37).

### Enrichment culturing and isolation of As(III)- and Sb(III)-oxidizing bacteria

Aerobic enrichment cultures were established by inoculating soil slurry in minimal Xm medium (50-mL medium in 160-mL serum bottles) (17) with 10 mM lactate for high carbon conditions (HCM) or 6 mM HCO_3_^−^ and 0.002% (wt/vol) yeast extract for low carbon conditions (LCM). The medium was amended with 100 μM Sb(III) (as potassium antimonyl tartrate) or 10 mM As(III) (as NaAsO_2_) and incubated at 25°C in the dark on a reciprocal shaker (120 min^−1^). The concentration of As(III) used in this study was selected based on the previous report which showed that the average minimum inhibitory concentration (MIC) of arsenite-resistant bacteria isolated from arsenic-contaminated soils ranged from 8 to14 mM (7). Although much less information was available regarding bacterial antimonite resistance levels, one report showed that antimonite concentrations of >200 μM strongly inhibit the growth of common soil bacteria (2); thus, we chose 100 μM Sb(III) for the enrichment culture in this study. Abiotic controls were prepared identically without inocula. Enrichment with HCM exhibited visible growth after a week and isolated colonies were obtained by plating serially diluted enrichment cultures on Xm plates prepared by the addition of purified agar (1.5% [wt/vol]) to Xm HCM media containing the same concentrations of Sb(III) or As(III) as the original enrichment. Colonies were randomly selected from plates inoculated with 10^−4^ and 10^−5^ dilutions of enrichment cultures and re-streaked for isolation. Since no visible growth was observed with LCM enrichment, Sb(III) or As(III) oxidation activities were monitored by inductively coupled plasma spectrometry (ICP-OES; PerkinElmer Optima 7300 DV; PerkinElmer) determination for total and pentavalent species after borohydride reduction-based liberation of Sb(III) and As(III) as stibine and arsine gases, respectively (24–26). Once oxidation occurred after 5 weeks, the enrichment cultures were serially diluted in fresh media and incubation continued under the same conditions. After multiple transfers, aliquots of the highest dilutions with oxidation activity [10^−8^ for both Sb(III) and As(III) LCM enrichments] were spread onto LCM Xm plates containing either Sb(III) or As(III). Colonies were re-streaked for isolation, then transferred to fresh liquid medium and incubated under the same conditions as the original enrichment. Once the purity of the cultures was verified, they were tested for the ability to oxidize Sb(III) or As(III) by growing with Xm medium (prepared with HPCL-grade water) in the presence of 100 μM Sb(III) or 10 mM As(III) for 7 d, followed by the determination of Sb(V) or As(V) production as described above. Cells were transferred at least three times under the same growth conditions prior to the activity assays. The activity assays were repeated at least twice and each time in duplicate.

### Characterization of *aioA* gene in As(III)-oxidizing isolate

The putative arsenite oxidzse gene, *aioA*, in the As(III)-oxidizing isolate obtained above was PCR amplified using aroA95f and aroA599r primers and the conditions described previously (19). To examine the expression of the *aioA* gene, the As(III)-oxidizing isolate was grown in the presence of As(III) (10, 1 and 0.1 mM) or 100 μM Sb(III) with 0.002% yeast extract, or yeast extract only. Cells were harvested at the late exponential phase and RNA was extracted using the RNeasy mini kit (Qiagen, Chatsworth, CA). Following extraction, RNA was treated with DNase using TURBO DNA-free kit (Ambion, Life Technologies, Grand Island, NY). RNA concentration was determined by absorption at 260 nm. RT-PCR was performed using the Access RT-PCR system (Promega, Madison, WI). The RT-PCR reaction mixture (50 μL) contained 1 μM of each primer and ~50 ng of extracted RNA. Control reactions were performed without the addition of reverse transcriptase to verify the absence of DNA in the RNA preparations. Amplified RT-PCR products were purified and sequenced to confirm the sequence identity of the transcripts.

### Nucleotide sequence accession number

The nucleotide sequences reported in this paper have been deposited in the GenBank database under accession numbers KC012938 to KC012943.

## Results and Discussions

### Site characterization

Chemical analysis showed elevated levels of As and Sb in soils from both site 1 and 2 (Table 1). At site 1, the concentrations of both As and Sb were highest in the surface layer (1,240 and 2,280 mg kg^−1^, respectively) and decreased with depth, which correlated also with the Fe and Mn concentrations (*R*_Fe_^2^= 0.97, *R*_Mn_^2^=0.93 for As; *R*_Fe_^2^=0.81, *R*_Mn_^2^=0.60 for Sb). This result indicated that the behaviors of Sb and As are associated with those of Fe and Mn in the soil profile. Previously, Mitsunobu *et al.* (29) conducted geochemical characterization of Ichinokawa mine soil when soil was under flooded conditions. It was shown that the concentrations of Sb and As increased slightly with depth (0~12 cm) and, consistent with our result, a positive correlation was observed with Fe and Mn. Further extended X-ray absorption fine structure (EXAFS) analyses for Fe and Mn suggested that the host phase of Sb and As is likely Fe(III) hydroxide at all depths in the soil profile (29); thus, the majority of As and Sb, originally supplied as sulfide minerals are present in labile forms. In contrast, As and Sb in site 2 were distributed rather evenly among 0–9 cm depths (130–160 and 1,400–1,500 mg kg^−1^, respectively). The concentrations of other constituents, including Mn, Cr, Ni, and Zn, were substantially higher in site 1 than site 2. These results suggest that site 1 soil, which is located downward from the mine pit, is still affected by the discharge of contaminated water from the mine pit.

Bacterial populations associated with the depth profiles of Ichinokawa soils were examined using PCR-amplified 16S rRNA gene fragments separated via DGGE (Fig. S1). No substantial changes in DGGE banding patterns were observed along the depths profile at both sites. Due to the high complexity of DGGE banding profiles, no serious attempts were made to identify DNA sequences of each band. However, the sequences of a couple of DGGE bands were determined and shown to be related to 16S rRNA gene sequences of uncultured bacterial clones obtained from soil environments, including heavy metal waste sites (data not shown).

To examine the presence of indigenous microbial populations possessing functional genes associated with arsenic transformations, soil DNA extracts were screened by PCR amplification using previously developed primer sets for arsenite oxidase gene (*aioA*) (19), anaerobic arsenite oxidase gene (*arxA*) (40), and dissimilatory arsenate reductase gene (*arrA*) (23, 27). Screening of soil DNA was limited to known functional genes associated with As transformations, since no information regarding the molecular mechanisms of prokaryotic Sb(III) oxidation is currently available. Positive PCR products were observed for *aioA* at all depths from site 1 and 2, indicating the potential presence of an aerobic arsenite-oxidizing population *in situ*, while no PCR products were obtained with any of the primer sets tested for *arxA* or *arrA* (Fig. S1).

### Enrichment culturing

To link indigenous microbial populations with their function in Sb and As oxidation, enrichment cultures were established aerobically using surface soils from site 1 and 2 as inocula (Table 2). The HCM (high carbon media) enrichment cultures, containing a minimal medium amended with 10 mM As(III) or 100 μM Sb(III) and 10 mM lactate as a carbon source, exhibited visible growth after one week. Isolated colonies were obtained from direct plating of HCM enrichment and randomly selected colonies were further identified by 16S rRNA gene sequencing. Although multiple colonies were isolated from HCM-As(III) enrichment with IK1-1 soil, they were unable to grow after subsequent inoculation into liquid media; thus, only the isolates obtained from IK2-1 enrichment were further characterized. All of the isolates obtained from HCM-Sb(III) enrichment were identified to be *Pseudomonas* spp., while *Stenotrophomonas*- and *Nocardia-*like isolates were obtained from HCM-As(III) enrichment (Table 2).

**Fig. 1:**
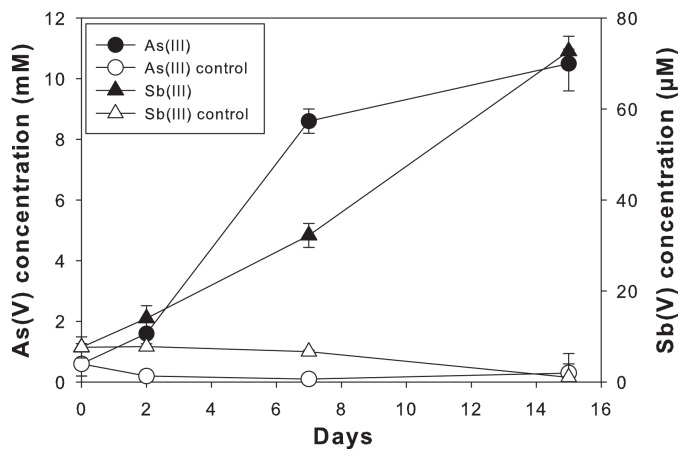
Oxidation of As(III) and Sb(III) by Ichinokawa soil enrichment cultures. Formation of As(V) in LCM IK1-1 enrichment (10^−8^ dilution from original enrichment) is shown in filled circle, formation of Sb(V) in LCM IK2-1 enrichment (10^−8^ dilution from original enrichment) is shown in filled triangle, and abiotic controls are shown in open symbols. Each point represents the mean of duplicate samples; error bars represent the standard error. Where absent, error bars are smaller than the symbol size.

**Fig. 2:**
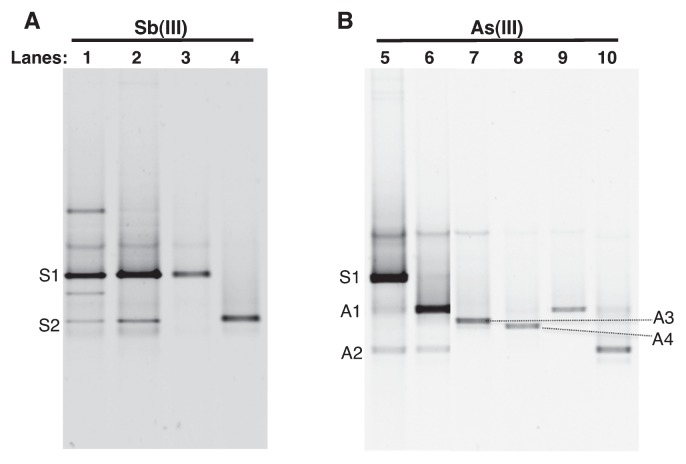
DGGE analysis of 16S rRNA gene fragments from enrichments and isolates obtained with (A) Sb(III) and (B) As(III) from Ichinokawa soil. Lanes: LCM IK2-1 Sb(III)-oxidizing consortia with 10^7^ (lane 1) and 10^8^ (lane 2) dilutions of the original enrichment, *Pseudomonas-*like S1 strain (lane 3), *Stenotrophomonas-*like S2 strain (lane 4), and LCM IK1-1 As(III)-oxidizing consortia with 10^7^ (lane 5) and 10^8^ (lane 6) dilutions of the original enrichment, *Stenotrophomonas-*like A3 strain (lane 7), *Nocardia-*like A4 strain (lane 8), *Stenotrophomonas-*like A1 strain (lane 9), *Sinorhizoium-*like A2 strain (lane 10). Letters indicate DGGE bands for which sequence data were obtained (Table 2).

Enrichment cultures were also established in a low carbon condition (LCM), containing 6 mM HCO_3_^−^ and 0.002% (wt/vol) yeast extract, amended with 10 mM As(III) or 100 μM Sb(III). LCM enrichment with As(III) inoculated with IK1-1 soil and Sb(III) inoculated with IK2-1 soil showed complete oxidation of added As(III) and Sb(III) after 6 weeks. Subsequently, these enrichments were serially diluted with fresh media and incubated further. After 30 d, two of the highest dilutions with oxidation activity (10^−7^ and 10^−8^ dilutions) were transferred again. As(III) and Sb(III) oxidation of 10^−8^ dilution cultures were monitored for 15 d (Fig. 1), which exhibited oxidation of 97.9±0.01% and 72.8±0.004% of added As(III) and Sb(III) compared to the abiotic controls, respectively. Bacterial populations present in these Sb- and As-oxidizing LCM enrichments (10^−7^ and 10^−8^ dilutions) were examined by 16S rRNA gene-targeted DGGE (Fig. 2). In the Sb(III)-oxidizing enrichment, two prominent DGGE bands, S1 and S2, were observed in 10^−8^ dilution (Fig. 2A, lane 2) while a few additional bands were present in the 10^−7^ dilution (Fig. 2A, lane 1). The same S1 band was also observed in As(III)-oxidizing enrichment with 10^−7^ dilution in addition to two unique A1 and A2 bands which were further enriched in 10^−8^ dilution (Fig. 2B, lane 5 and 6).

### Isolation of Sb(III)- and As(III)-oxidizing bacteria

The dominant phylotypes detected via DGGE in As(III)-and Sb(III)-oxidizing LCM enrichments were further isolated by plating and re-streaking morphologically distinct colony types (Table 2 and Fig. 2). *Pseudomonas-*like isolates obtained from Sb(III) LCM enrichment corresponded to S1 bands and were also identical to eight *Pseudomonas* isolates obtained from Sb(III) HCM enrichments. *Stenotrophomonas*-like isolates obtained from Sb(III) LCM enrichment corresponded to S2 bands and were also identical to four *Stenotrophomonas* isolates (=DGGE band A3) obtained from As(III) HCM enrichment (Table 2 and Fig. 2). From As(III) LCM enrichment, *Stenotrophomonas-* and *Sinorhizobium*-like isolates corresponding to A1 and A2 bands (Table 2 and Fig. 2B), respectively, were obtained and were distinct from other isolates obtained from HCM enrichments.

Six representative strains isolated from the enrichment cultures were examined for their As(III)- and Sb(III)-oxidizing activities. Cultures were inoculated into a basal medium in the presence of As(III) or Sb(III) with different carbon source conditions (HCMy: lactate+0.002% yeast, LCM: 0.002% yeast), and the formation of As(V) and Sb(V) was determined after 7-day incubation (Table 3). All six isolates grew comparably either in the presence or absence of 100 μM Sb(III) in HCMy (average final cell densities of 1.7–2.1×10^8^ cells per ml). *Pseudomonas-*like strain S1 and *Stenotrophomonas-*like strain A3 oxidized approximately 18% and 8% of added Sb(III), respectively, during growth to the stationary phase (7 d) compared to the abiotic controls (*P*<0.05). Further oxidation was not observed by extending the incubation period to 2 weeks. A previous study showed that *A. tumefaciens* culture amended with 50 μM Sb(III) produced ~10 μM Sb(V) during growth to the stationary phase (~35 h) (25). In our study, an initial concentration of 100 μM Sb(III) was added to the cultures and 8–18 μM was oxidized to Sb(V) when cells reached the early stationary phase (7 d). Although the rates of Sb(III) oxidation by strains S1 and A3 were substantially slower than that of *A. tumefaciens*, total amounts of Sb(III) oxidized during growth were comparable among these isolates. Strain S2 originally showed Sb(III) oxidation activity comparable to strain A3; however, the activity was unstable and decreased over the course of transfers. Strain S1 was also able to grow in LCM and oxidize ~18 μM of Sb(III), while strain A3 did not grow in LCM. Sb(III)-oxidizing activities of the obtained isolates were substantially lower than that of the original consortium where ~73 μM of Sb(III) was oxidized in 2 weeks (Fig. 1). It is possible that the Sb(III) oxidation process may involve multiple organisms in the consortium, or other minor populations in the consortium which were not isolated here may play important roles in Sb(III) oxidation.

Among the six isolates, only *Sinorhizobium*-like strain A2 showed As(III) oxidation activity and oxidized 10 and 8.7 mM of As(III) to As(V) during growth in HCMy and LCM media, respectively (Table 3). As(III) oxidation was not observed without cells or with autoclaved cells of strain A2 under the same growth conditions, confirming the biotic oxidation of As(III). Strain A2 grew comparably in LCM with or without As(III), and it was not clear if energy was gained from As(III) oxidation during growth, as the final cell density of strain A2 was similar when As(III) was present compared to its absence.

### Phylogenetic characterization of Sb(III)- and As(III)-oxidizing bacteria

**Fig. 3:**
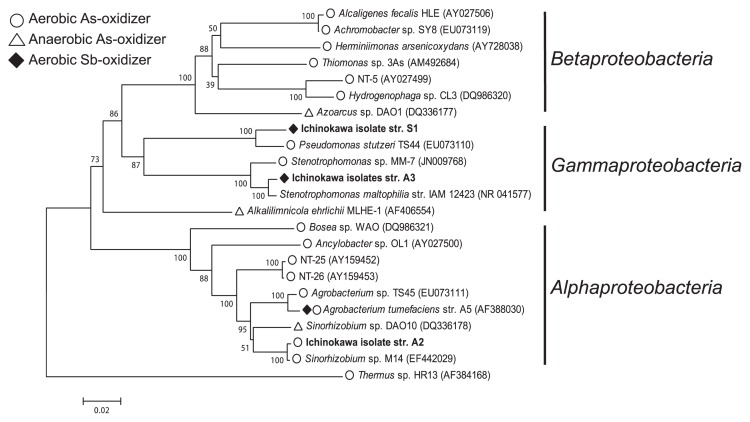
Neighbor-joining tree showing phylogenetic positions of 16S rRNA gene sequences of Sb(III)- and As(III)-oxidizing isolates obtained from Ichinokawa soil (shown in black bold). Open circle, aerobic As(III)-oxidizing bacteria; open triangle, anaerobic As(III)-oxidizing bacteria; closed diamond, aerobic Sb(III)-oxidizing bacteria. Bootstrap values (per 1,000 trails) for major branch points are indicated. Bar=0.02 substitutions per sequence position.

A phylogenetic tree based on 16S rRNA gene sequences was constructed to compare the sequences of strains S1, A2 and A3 with other known Sb(III) and As(III) oxidizers (Fig. 3). Sb(III)-oxidizing strains were both affiliated with *Gammaproteobacteria* (Fig. 3). Strain S1 was closely related (97.1% 16S rRNA gene sequence identity) to an arsenite-oxidizing heterotroph, *P. stutzeri* TS44, isolated from arsenic-contaminated soil in China (7). Strain A3 belonged to the genus *Stenotrophomonas*, which has often been observed in association with high metalloid-containing environments, including arsenic-contaminated soils (5, 7), arsenic-enrichment cultures (35, 36), and a selenite-contaminated soil (4). Some of the *Stenotrophomonas* strains isolated from such environments exhibited resistance to high concentrations of metalloids (*i.e.*, As and Se). Recently, an arsenite-oxidizing *Stenotrophomonas* sp. MM-7 was also isolated (5), which showed 97.4% 16S rRNA gene sequence identity to strain A3; however, Sb(III) oxidation by this strain has not been examined. Our study is the first report of Sb(III) oxidation by *Stenotrophomonas* and *Pseudomonas* isolates.

As(III)-oxidizing strain A2 was affiliated with the Rhizobiaceae family of *Alphaproteobacteria*, and closely related (99.6% sequence identity) to *Sinorhizobium* sp. M14, a psychrotolerant arsenite oxidizer previously isolated from a gold mine in Poland (9). *Sinorhizobium* sp. M14 was shown to grow autotrophically using arsenite as an electron donor and inorganic carbon as a carbon source (9). Previous studies showed that the presence of yeast extracts (0.004–0.04%) stimulated the rate of growth and arsenite oxidation by facultative chemolithotrophic As(III) oxidizers belonging to the *Alphaproteobacteria*, including *Sinorhizoium* sp. M14 (9), strain NT-26 (33), and *Ancylobacter dichloromethanicus* As3-1b (3). Although the autotrophic growth of strain A2 coupled with As(III) oxidation was not confirmed in this study, As(III)-oxidizing bacteria in general seem to have a versatile metabolism and are able to utilize both organic and inorganic substrates as energy sources.

### Arsenite oxidase gene

**Fig. 4:**
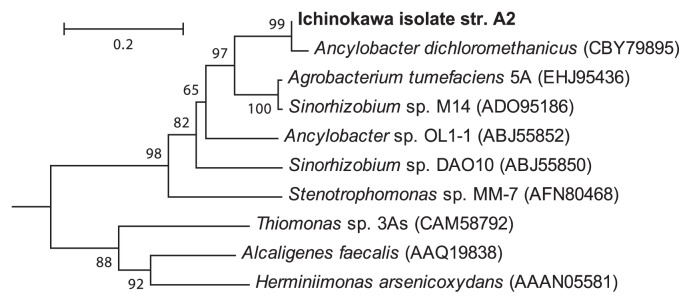
Phylogenetic position of the deduced amino acid sequence encoded by the putative arsenite oxidase gene (*aio*A) from As(III)-oxidizing Ichinokawa isolate strain A2. Tree=Neighbor-joining method; bar=0.2 substitutions/sequence position; Bootstrap values (per 1,000 trials) greater than 50% are indicated. Aio from *Thermus* sp. HR13 (ABB17183) was used as an out-group (not shown).

Aerobic arsenite oxidase gene (*aioA*) was identified in As(III)-oxidizing strain A2. Phylogenetic analysis of the deduced amino acid sequence encoded by the *aioA-*like sequence of strain A2 (Fig. 4) showed that it clustered with other AioA from arsenite-oxidizing *Alphaproteobacteria*. Although 16S rRNA gene sequence from strain A2 was most closely related to that of *Sinorhizobium* sp. M14, the deduced amino acid sequence of *aioA* was more closely related (99.4% aaID) to AioA from a facultative chemolithotrophic As(III) oxidizer, *A. dichloromethanicum* (3) than *Sinorhizobium* sp. M14 (90.0% aaID). Recent studies have also shown inconsistencies between 16S rRNA and AioA phylogenetic tree topologies, suggesting the potential role of horizontal gene transfer in the propagation of *aio* genes (3, 20, 30).

The expression of the *aioA* gene was confirmed in strain A2 during growth in the presence of As(III) (10 mM and 1 mM) or 100 μM Sb(III) using RT-PCR, while cells grown in the absence of As(III) did not express the *aioA* gene (Fig. S2). The induction of *aio* gene expression by As(III) and Sb(III) was also reported with As(III)- and Sb(III)-oxidizing *A. tumefaciense* (25), as well as As(III)-oxidizing *Achromobacter* sp. SY8 and *Pseudomonas* sp. TS44 (8). The regulatory protein coded by the *aioR* gene in *aio* operon is a member of the two-component signal transduction system involved in the regulation of As(III) oxidation (22) and this *aio* gene regulatory system seems to respond to both As(III) and Sb(III) interchangeably due to their similar properties. The fact that strain A2 was unable to oxidize Sb(III) when the *aioA* gene was expressed indicates the involvement of different mechanisms for the oxidation of arsenite and antimonite, as previously shown with a heterotrophic arsenite and antimonite-oxidizing *A. tumefaciens* strain A5 (25).

In conclusion, our results showed the presence of As(III)- and Sb(III)-oxidizing bacteria associated with highly contaminated mine tailings, and these indigenous microorganisms possibly contribute to the speciation and mobility of Sb and As *in situ*. This study expanded the list of the under-studied group of microorganisms having the ability to oxidize Sb(III), presenting an opportunity for further investigations of microbial roles in the Sb biogeochemical cycle.

## Supplementary Material



## Figures and Tables

**Table 1:** Concentrations of chemical constituents in Ichinokawa mine soils

	Sample ID	Depth (cm)	Concentration (mg kg^−1^)									

			As	Sb	Se	Pb	Fe	Mn	Cr	Ni	Cu	Zn
Site 1	IK1-1	0–3	1,240	2,280	16	20	43,100	12,800	123	105	61	631
	IK1-2	3–6	564	2,280	18	18	34,300	7,890	158	110	42	132
	IK1-3	6–9	175	499	18	16	32,000	3,910	148	119	40	518
	IK1-4	9–12	133	330	15	18	30,100	2,240	143	87	34	380
Site 2	IK2-1	0–3	128	1,470	15	29	23,500	659	42	40	51	146
	IK2-2	3–6	159	1,500	18	37	25,300	922	46	44	50	112
	IK2-3	6–9	138	1,400	13	37	24,300	976	48	43	51	111
	IK2-4	9–12	282	2,470	22	41	22,500	923	47	46	53	111

**Table 2:** Summary of enrichment conditions and obtained isolates in this study

Enrichment conditions		Inoculum	Growth^c^	Isolation method^d^	No. of isolates	DGGE band^e^	Closest GenBank relative (accession number) %	Identity
HCM^a^	As(III)	IK1-1	+++	DP	0	NA^f^	NA	
		IK2-1	+++	DP	4	A3=S2	*Stenotrophomonas maltophilia* str.6B2-1 (AY445079)	99.8
					1	A4	*Nocardia globerula* (AF430065)	99.0
	Sb(III)	IK1-1	+++	DP	5	S1	*Pseudomonas putida* (AF094742)	99.4
		IK2-1	+++	DP	3	S1	*P. putida* (AF094742)	99.6
LCM^b^	As(III)	IK1-1	+	SD	5	A1	*S. maltophilia* str. LMG10857 (AJ131117)	99.8
					1	A2	*Sinorhizobium morelense* str. LMG21331 (AM181737)	99.8
	Sb(III)	IK2-1	+	SD	6	S1	*P. putida* (AF094742)	99.6
					2	S2	*S. maltophilia* str.6B2-1 (AY445079)	99.7

a HCM: high carbon media containing 10 mM lactate.

b LCM: low carbon media containing 6 mM HCO_3_^−^ and 0.002% yeast extract

c Growth was determined by measuring OD600, +: 0.007–0.015, ++: 0.015–0.1, +++: >0.1

d DP, isolation by direct plating of enrichments; SD: isolation by serial dilution of enrichments followed by plating.

e DGGE bands correspond to Fig. 2.

f NA, not applicable.

**Table 3:** Sb(III) and As(III) oxidation activities of representative isolates obtained from Ichinokawa soil

Condition^a^		Concentrations of Sb(V) produced during 7-day incubation (μM)						

		Strain S1	Strain S2	Strain A1	Strain A2	Strain A3	Strain A4	Control^e^
Sb(III) 100 μM	HCMy	28.0±8.7*	14.1±4.7	BD^b^	BD	17.5±1.8*	BD	9.6±0.9
	LCM	28.0±0.7*	11.4±1.8	—c	—	NG^d^	—	10.5±0.6

Concentrations of As(V) produced during 7-day incubation (mM)								

As(III) 10 mM	HCMy	BD	BD	BD	10.3±0.9*	BD	BD	0.2±0.0
	LCM	—	—	—	8.7±0.3*	—	—	0.1±0.0

a HCMy: 10 mM lactate+0.002% (w/v) yeast extract, LCM: 6 mM HCO_3_^−^ and 0.002% yeast extract.

b BD: below detection limit or the abiotic control.

c —: not determined.

d NG: no growth.

e Control: abiotic controls.

* *P*<0.05 vs. abiotic controls (Student’s *t*-test).

## References

[1] Altschul SF, Gish W, Miller W, Myers EW, Lipman DJ (1990). Basic local alignment search tool. J Mol Biol.

[2] An Y-J, Kim M (2009). Effect of antimony on the microbial growth and the activities of soil enzymes. Chemosphere.

[3] Andreoni V, Zanchi R, Cavalca L, Corsini A, Romagnoli C, Canzi E (2012). Arsenite oxidation in *Ancylobacter dichloromethanicus* As3-1b strain: detection of genes involved in arsenite oxidation and CO_2_fixation. Curr Microbiol.

[4] Antonioli P, Lampis S, Chesini I, Vallini G, Rinalducci S, Zolla L, Righetti PG (2007). *Stenotrophomonas maltophilia* SeITE02, a new bacterial strain suitable for bioremediation of selenite-contaminated environmental matrices. Appl Environ Microbiol.

[5] Bahar MM, Megharaj M, Naidu R (2012). Arsenic bioremediation potential of a new arsenite-oxidizing bacterium *Stenotrophomonas* sp. MM-7 isolated from soil. Biodegradation.

[6] Bancroft P (1988). Famous mineral localities: the Ichinokawa mine. Mineral Rec.

[7] Cai L, Liu G, Rensing C, Wang G (2009). Genes involved in arsenic transformation and resistance associated with different levels of arsenic-contaminated soils. BMC Microbiol.

[8] Cai L, Rensing C, Li X, Wang G (2009). Novel gene clusters involved in arsenite oxidation and resistance in two arsenite oxidizers: *Achromobacter* sp. SY8 and *Pseudomonas* sp. TS44. Appl Microbiol Biotechnol.

[9] Drewniak L, Matlakowska R, Sklodowska A (2008). Arsenite and arsenate metabolism of *Sinorhizobium* sp. M14 living in the extreme environment of the Zloty Stok gold mine. Geomicrobiol J.

[10] Duquesne K, Lieutaud A, Ratouchniak J, Muller D, Lett M-C, Bonnefoy V (2008). Arsenite oxidation by a chemoautotrophic moderately acidophilic *Thiomonas* sp.: from the strain isolation to the gene study. Environ Microbiol.

[11] Ehrlich HL, Newman DK, Ehrlich HL, Newman DK 200Geomicrobial interactions with arsenic and antimony. Geomicrobiology.

[12] Ferris MJ, Muyzer G, Ward DM (1996). Denaturing gradient gel electrophoresis profiles of 16S rRNA-defined populations inhabiting a hot spring microbial mat community. Appl Environ Microbiol.

[13] Filella M, Belzile N, Chen Y-W (2002). Antimony in the environment: a review focused on natural waters I. Occurrence Earth Sci Rev.

[14] Filella M, Belzile N, Chen Y-W (2002). Antimony in the environment: a review focused on natural waters II. Relevant solution chemistry. Earth Sci Rev.

[15] Fu H-L, Rosen BP, Bhattacharjee H (2010). Biochemical characterization of a novel ArsA ATPase complex from Alkaliphilus metalliredigens QYMF. FEBS Letters.

[16] Ha NN, Agusa T, Ramu K (2009). Contamination by trace elements at e-waste recycling sites in Bangalore, India. Chemosphere.

[17] Hamamura N, Storfa RT, Semprini L, Arp DJ (1999). Diversity in butane monooxygenases among butane-grown bacteria. Appl Environ Microbiol.

[18] Hamamura N, Olson SH, Ward DM, Inskeep WP (2005). Diversity and functional analysis of bacterial communities associated with natural hydrocarbon seeps in acidic soils at Rainbow Springs, Yellowstone National Park. Appl Environ Microbiol.

[19] Hamamura N, Macur RE, Korf S, Ackerman GG, Taylor WP, Kozubal M, Reysenbach A-L, Inskeep WP (2009). Linking microbial oxidation of arsenic with detection and phylogenetic analysis of arsenite oxidase genes in diverse geothermal environments. Environ Microbiol.

[20] Heinrich-Salmeron A, Cordi A, Brochier-Armanet C (2011). Unsuspected Diversity of Arsenite-oxidizing bacteria as revealed by widespread distribution of the *aox* B gene in prokaryotes. Appl Environ Microbiol.

[21] Ji G, Garber EAE, Armes LG, Chen C, Fuchs JA, Silver S (1994). Arsenate reductase of *Staphylococcus aureus* Plasmid pI258. Biochemistry.

[22] Kashyap DR, Botero LM, Franck WL, Hassett DJ, McDermott TR (2006). Complex regulation of arsenite oxidation in *Agrobacterium tumefaciens*. J Bacteriol.

[23] Kulp TR, Hoeft SE, Miller LG, Saltikov C, Murphy JN, Han S, Lanoil B, Oremland RS (2006). Dissimilatory arsenate and sulfate reduction in sediments of two hypersaline, arsenic-rich soda lakes: Mono and Searles lakes, California. Appl Environ Microbiol.

[24] Langner HW, Jackson CR, McDermott TR, Inskeep WP (2001). Rapid oxidation of arsenite in a hot spring ecosystem, Yellowstone National Park. Environ Sci Technol.

[25] Lehr CR, Kashyap DR, McDermott TR (2007). New insights into microbial oxidation of antimony and arsenic. Appl Environ Microbiol.

[26] Macur RE, Jackson CR, Botero LM, McDermott TR, Inskeep WP (2004). Bacterial populations associated with the oxidation and reduction of arsenic in an unsaturated soil. Environ Sci Technol.

[27] Malasarn D, Saltikov CW, Campbell KM, Santini JM, Hering JG, Newman DK (2004). arrA is a reliable marker for As(V) respiration. Science.

[28] Meng Y-L, Liu Z, Rosen BP (2004). As(III) and Sb(III) uptake by GlpF and efflux by ArsB in *Escherichia coli*. J Biol Chem.

[29] Mitsunobu S, Harada T, Takahashi Y (2006). Comparison of antimony behavior with that of arsenic under various soil redox conditions. Environ Sci Technol.

[30] Quemeneur M, Heinrich-Salmeron A, Muller D, Lievremont D, Jauzein M, Bertin PN, Garrido F, Joulian C (2008). Diversity surveys and evolutionary relationships of *aoxB* genes in aerobic arsenite-oxidizing bacteria. Appl Environ Microbiol.

[31] Rhine ED, Garcia-Dominguez E, Phelps CD, Young LY (2005). Environmental microbes can speciate and cycle arsenic. Environ Sci Technol.

[32] Sanders OI, Rensing C, Kuroda M, Mitra B, Rosen BP (1997). Antimonite is accumulated by the glycerol facilitator GlpF in *Escherichia coli*. J Bacteriol.

[33] Santini JM, Sly LI, Schnagl RD, Macy JM (2000). A new chemolithoautotrophic arsenite-oxidizing bacterium isolated from a gold mine: phylogenetic, physiological, and preliminary biochemical studies. Appl Environ Microbiol.

[34] Stolz JF, Basu P, Oremland RS (2010). Microbial arsenic metabolism: new twists on an old poison. Microbe.

[35] Sun W, Sierra-Alvarez R, Milner L, Field JA (2010). Anaerobic oxidation of arsenite linked to chlorate reduction. Appl Environ Microbiol.

[36] Sun WJ, Sierra-Alvarez R, Fernandez N, Sanz JL, Amils R, Legatzki A, Maier RM, Field JA (2009). Molecular characterization and in situ quantification of anoxic arsenite-oxidizing denitrifying enrichment cultures. FEMS Microbiol Ecol.

[37] Tamura K, Peterson D, Peterson N, Stecher G, Nei M, Kumar S (2011). MEGA5: Molecular evolutionary genetics analysis using maximum likelihood, evolutionary distance, and maximum parsimony methods. Mol Biol Evol.

[38] Yang N, Sun H, Sun H (2011). Biological chemistry of antimony and bismuth. Biological chemistry of arsenic, antimony and bismuth.

[39] Zargar K, Hoeft S, Oremland R, Saltikov CW (2010). Identification of a novel arsenite oxidase gene, *arxA*, in the haloalkaliphilic, arsenite-oxidizing bacterium *Alkalilimnicola ehrlichii* strain MLHE-1. J Bacteriol.

[40] Zargar K, Conrad A, Bernick DL, Lowe TM, Stolc V, Hoeft S, Oremland RS, Stolz J, Saltikov CW (2012). ArxA, a new clade of arsenite oxidase within the DMSO reductase family of molybdenum oxidoreductases. Environ Microbiol.

