# Supplementary material for: Identification of Antimony- and Arsenic-Oxidizing Bacteria Associated with Antimony Mine Tailing
Source: Microbes Environ. 2013 May 11;28(2):257–63. doi: 10.1264/jsme2.ME12217 (PMC4070671; doi:10.1264/jsme2.ME12217)

Supplemental Online Material:

Title: Identification of antimony- and arsenic-oxidizing bacteria associated with antimony mine tailings

Authors: Hamamura, N., Fukushima, K., Itai, T.

Supplemental figure S1. DGGE profiles of 16S rRNA gene fragments and the presence of arsenic transformation functional genes along the depth profile of Ichinokawa mine soils. Sample IDs correspond the description in Table1. The presence of functional genes associated with arsenite oxidation (*aio* and *arx*) and arsenate reduction (*arr*) was examined by using PCR as described in Materials and Methods and results are shown as: -, no amplification; +, weak amplification; ++, strong amplification.

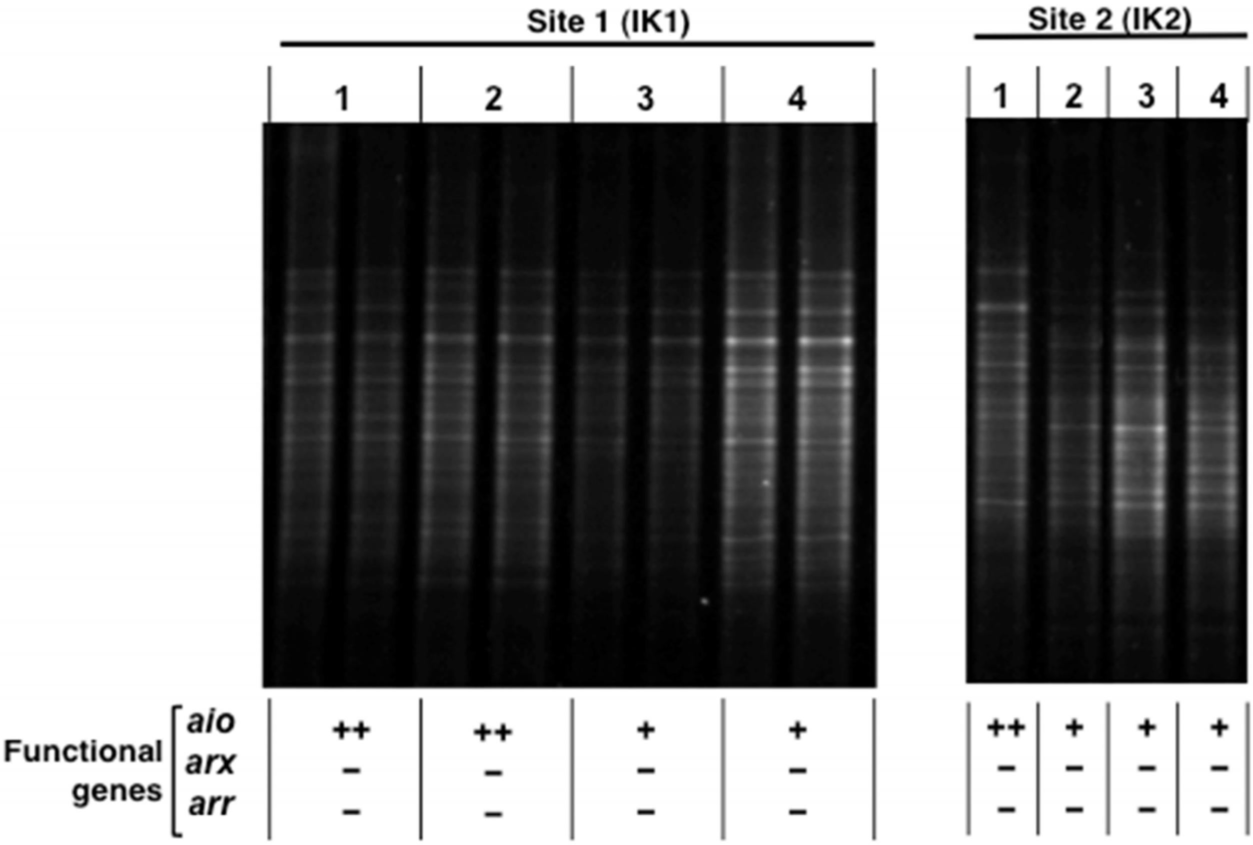

Supplemental figure S2. Detection of *aioA* transcripts in strain A2 growing in the presence of 0.1, 1, or 10 mM As(III), and 100 μM Sb(III).

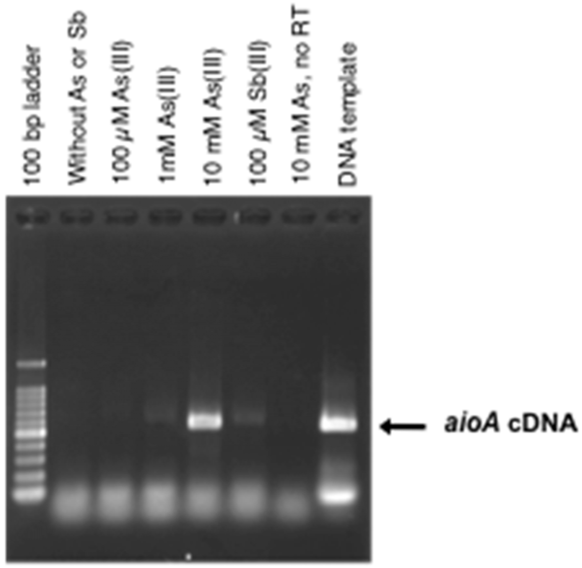

Supplement: Supplementary file 1 [file 28_257_s1.pdf]
